# Enhanced immunogenicity of a nanoparticle therapeutic cancer vaccine targeting HAAH delivered intradermally using 3M's hollow microstructured transdermal system (hMTS)

**DOI:** 10.1186/2051-1426-3-S2-P433

**Published:** 2015-11-04

**Authors:** Steven Fuller, Michael Lebowitz, Solomon Stewart, Susan Walker, Hossein Ghanbari, Allan Bohlke, Scott Burton, Leonard Chu, Vinh Hua

**Affiliations:** 1 Panacea Pharmaceuticals, Inc., Gaithersburg, MD, USA; 2 3M Company, St. Paul, MN, USA

## Background

We are evaluating the immunogenicity and efficacy of a nanoparticle vaccine (NPV) targeting the tumor marker human aspartyl (asparaginyl) β-hydroxylase (HAAH). HAAH is an embryonic protein that is over-expressed on the surface of cancer cells, is demonstrated to be responsible for cell proliferation, motility and invasiveness, processes which can be inhibited by anti-HAAH antibodies *in vitro*. We have developed novel anticancer NPVs in which portions of the HAAH molecule are expressed on λ-phage (200-300 copies per NPV). These NPVs produce high-titer anti-HAAH polyclonal antibodies in mice despite the fact that the HAAH protein is highly conserved between mammalian species. We have shown that the NPV inhibits tumor growth and metastasis in mouse liver and breast cancer models and inhibits metastasis in a rat prostate cancer model, inducing both humoral and cellular responses. We report here the use of 3M's hollow microstructured transdermal system (hMTS) to determine if this intradermal (ID) delivery device further enhances immunogenicity of the NPV.

## Methods

Intramuscular (IM) immunization of the NPV was compared with ID administration using the 3M hMTS device. Male Sprague-Dawley rats (N=6/group) were immunized with 2.5x10^11^ phage particles displaying the N-terminal (HAAH-1λ) third of HAAH. Two groups received one injection either IM via syringe or ID via hMTS on Day 0, while two additional groups received IM or ID injections on Days 0, 14 and 28. Repeat injections using the hMTS device were made to the same skin site on the dorsal thoracic region.

## Results

The vaccine was well tolerated in all groups. No local injection site or systemic adverse effects were observed in any animal. Blood samples were obtained on Days 0, 14, 28 and 35 for assessment of antibody levels by ELISA on recombinant HAAH- or H460 human lung cancer cell-coated plates. The Day 14 antibody titers were similar in all groups, while in the two groups that received three doses of vaccine, the antibody titers were boosted after each additional injection. Importantly, the Day 35 antibody titers were much higher using the ID versus IM immunization when tested on both antigens (rHAAH coating: 5,777 ID vs. 1,392 IM, p

## Conclusion

These data provide evidence that ID delivery of NPV targeting HAAH using the hMTS device is more effective than traditional IM delivery in inducing an immune response. We are preparing an IND to conduct clinical studies.

